# The role of fire in UK upland management: the need for informed challenge to conventional wisdoms: a comment on Davies *et al.* (2016)

**DOI:** 10.1098/rstb.2016.0433

**Published:** 2016-11-19

**Authors:** David J. T. Douglas, Graeme M. Buchanan, Patrick Thompson, Jeremy D. Wilson

**Affiliations:** 1 RSPB Centre for Conservation Science, RSPB Scotland, 2 Lochside View, Edinburgh Park, Edinburgh EH12 9DH, UK; 2 RSPB, 1 Sirius House, Amethyst Road, Newcastle Business Park, Newcastle-upon-Tyne NE4 7YL, UK

Davies *et al.* [1] argue that prescribed burning is an important ecological management tool with deep, historical roots and that debate about the role of fire in management of the UK uplands should be informed and unbiased. We agree on both counts. The Royal Society for the Protection of Birds' (RSPB's) prescribed burning experiments for managing native pinewoods for capercaillie *Tetrao urogallus* [2] exemplify this approach, informing our use of this form of prescribed burning. Davies *et al.* also argue that informed and unbiased debate on burning is threatened by a trend of ‘simplifying the narrative’ in the scientific literature and media to present fire as ‘only ecologically damaging’. In doing so, they criticize the scientific interpretation and media communication of RSPB [3] and academic research which they consider fails to meet their standards. Regrettably, Davies *et al.* model many of the behaviours of which they accuse others. We illustrate this below, correct scientific misrepresentations of our work by Davies *et al.* and challenge the view that the current narrative is over-simplified or insufficient to inform policy.

Davies *et al.* [1] question allegedly selective citation practices of other scientists, but fail to meet their own standards. For example, in a previous discussion of seasonal timing of prescribed burning and wildfire on moorland, Davies *et al.* [4] state ‘Early spring is the peak period for wildfire activity in the UK. Legg *et al.* (2007)’, yet in [1], they state ‘Management fires are set in winter or early spring … By contrast, wildfires predominantly occur in spring and summer … (Legg *et al.* 2007)’. A shift in position based on new evidence would be one thing, but use of the same citation to support two different statements on the timing of wildfires is quite another.

Regarding scientific criticisms and uses of Douglas *et al.* [3] that require correction, Davies *et al.* [1] firstly claim that Douglas *et al.* [3,5] misinterpret MODIS fire detections and confuse total area burned of prescribed fires with fire front area (relevant to fire size detectable by MODIS). This is incorrect. Douglas *et al.* [5] calculated fire detection probability as a function of measured length of burn scar and width of fire front (c1–3 m). An alternative would be measurement of burn widths to recalculate fire front area. However, this does not change the dataset of MODIS fire detections which, in areas managed for red grouse *Lagopus lagopus scotica* shooting, show a striking seasonal correspondence with the prescribed burning season [5]. Based on a conviction that prescribed burns are too small for MODIS detection, Davies *et al.* [4] state that MODIS fire detections must be wildfires. So, either MODIS detects a proportion of prescribed burns and serves as an index of temporal trends in burning activity (our interpretation) or, within areas managed for red grouse, wildfires are inextricably linked to grouse moor burning and are increasing in frequency, raising questions about the role of prescribed burning as an ignition source of wildfires. We agree with Davies *et al.* [1] that the role of managed moorland burning in protecting against future wildfires is unverified.

Secondly, the use by Davies *et al.* of values in [3] to estimate burning rotation lengths and annual areas burned is inappropriate. Data in [3] are derived from one image per 1 square kilometre, preventing estimation of repeat burning times, as only the most recent scar from re-burned patches will be visible. This is crucial as Allen *et al.* [6] report that 59% of burned area at their study commencement was burned at least twice during the c20 year study. Furthermore, average fire return times to burned patches were 16.1 years in the 2000s in [7], also within the c25 year timespan of [3]. Davies *et al.* [1] estimate percentage areas of moorland burned annually, using [3], of 0.04–3.8% ‘on individual moors’. The calculation methods are not explained but we assume it uses percentage areas burned per 1 square kilometre. As above, these are inappropriate for calculating *annual* areas burned. Disregarding of repeat burning renders all the associated calculations in [1] grossly misleading.

Davies *et al.* judge that press releases associated with [3] were not a fair reflection of its key findings. Their judgement relies on a student perception exercise, but their presentation of reproducible methods fails to meet minimum requirements, detracting from their goal of informed debate. We do note though that text in one press release stating that the study ‘ … revealed the extent of moorland burning across Britain's upland areas *and the damage it can cause*’ (our italics) does, in the italicized text, exceed the research findings, and we acknowledge that these words should not have been included. However, given Davies *et al.*'s advice that “…authors ensure that the press releases associated with their findings accurately reflect the content of their research…”, it is unfortunate that their own accepted manuscript was the subject of media coverage (e.g. [8]) prior to publication which resulted in public criticism of RSPB based on wording which did not appear in the published version of [1].

In perhaps the key point, Davies *et al.* [1] suggest that evidence for environmental impacts of moorland burning is insufficient to formulate policies for sustainable management. We agree that this evidence base is incomplete, but the reviews highlighted by Davies *et al.* [1] collectively contain evidence of negative impacts of burning on site habitat condition, carbon storage, drinking water quality and aquatic biodiversity. These are issues of fundamental societal concern and it is reasonable to consider the precautionary principle when addressing management that impacts on these ecosystem services. A systematic review [9] similarly concluded ‘Pending further research it is suggested that burning on blanket bog and wet heath should normally be avoided if favourable condition is to be achieved or maintained’. Furthermore, Harris *et al.* [10] state ‘many moorlands in upland Britain occur on peaty soils that should sequester carbon and many are also drinking water catchments, where a reduced water quality (increased coloration) will increase purification costs. For these latter ecosystem services, a reduction in burning frequency or a no-burn policy would be advised’. Policy-makers may consider that public benefits including biodiversity conservation, carbon storage and sequestration and provision of drinking water should not be traded-off against the private production of red grouse for sport shooting, of which rotational strip burning is an integral management, and this is a legitimate challenge [11]. Davies *et al.*'s call for landscape-scale moorland studies incorporating burning is admirable, though not new (e.g. [12]), but such calls have yet to attract significant funding. Until then, decisions regarding sustainable upland management still need to be taken, using the best available evidence, without the luxury of meeting all the evidence needs in [1].

Throughout their paper, Davies *et al.* premise their argument on the importance of burning for managing the UK uplands, and conclude by stating ‘ … to retain moorlands and peatlands as one part of a diversity of upland landscape structures, fire will need to be part of their management’. Given that burning in the UK uplands can have negative environmental impacts (see above), and the manifest societal and environmental benefits of restoring peat-dominated ecosystems [13], a more critical approach to the use of burning in the UK uplands would be prudent. Questions requiring prompt attention are (i) what role does burning have in environmentally sustainable upland management and (ii) is peatland restoration aided or hindered by burning? Addressing these questions may challenge some deeply rooted conventional wisdoms but will help to ensure that debate is genuinely informed and unbiased.
